# Supplementary material for: Coffee Silverskin as a Functional Ingredient in Vegan Biscuits: Physicochemical and Sensory Properties and In Vitro Bioaccessibility of Bioactive Compounds
Source: Foods. 2022 Feb 28;11(5):717. doi: 10.3390/foods11050717 (PMC8909313; doi:10.3390/foods11050717)
Supplement: Supplementary file 1 [file foods-11-00717-s001.zip › foods-1594200-supplementary.pdf]

foods

Supplementary Material

Coffee Silverskin as Functional Ingredient in Vegan Biscuits: Physicochemical and Sensory Properties and In Vitro Bioaccessibility of Bioactive Compounds

Carolina Cantele [1], Martina Tedesco [1], Daniela Ghirardello[1], Giuseppe Zeppa[1] and Marta Bertolino [1,*]

[1] Department of Agricultural, Forest and Food Sciences (DISAFA), University of Turin, 10095 Grugliasco, Italy; carolina.cantele@unito.it (C.C.); martina.tedesco@unito.it (M.T.); daniela.ghirardello@unito.it (D.G.); giuseppe.zeppa@unito.it (G.Z.)

* Correspondence: marta.bertolino@unito.it; Tel.: +39 0116708686

Keywords: coffee silverskin; decaffeination; by-products; polyphenols; in vitro digestion; bioaccessibility; antiradical activity

Publisher's Note: MDPI stays neutral with regard to jurisdictional claims in published maps and institutional affiliations.

Copyright: © 2022 by the authors. Licensee MDPI, Basel, Switzerland. This article is an open access article distributed under the terms and conditions of the Creative Commons Attribution (CC BY) license (https://creativecommons.org/licenses/by/4.0/).

Table S1. Specific levels of the 7-point hedonic scale used for the purchase predisposition parameter of the consumer acceptance test.

Level	*Descriptor*
1	definitely no
2	no
3	probably no
4	neither yes nor no
5	probably yes
6	yes
7	definitely yes

Figure S1. Visual aspect of the three types of coffee silverskin (CS) used to produce the biscuits. From left to right: Arabica CS, Robusta CS, and decaffeinated CS.

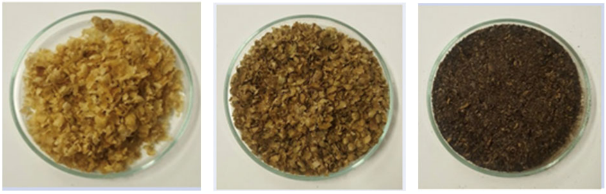

Table S2. Values of ΔE calculated among the coffee silverskin-added biscuits, using the CIELAB values.

Comparison	ΔE
0CS *vs.* 2CSA	4.19
0CS *vs.* 4CSA	7.31
0CS *vs.* 6CSA	10.44
0CS *vs.* 2CSR	9.06
0CS *vs.* 4CSR	15.58
0CS *vs.* 6CSR	20.39
0CS *vs.* 2CSD	15.57
0CS *vs.* 4CSD	24.58
0CS *vs.* 6CSD	29.47
2CSA *vs.* 4CSA	3.39
4CSA *vs.* 6CSA	3.13
2CSR *vs.* 4CSR	6.75
4CSR *vs.* 6CSR	4.99
2CSD *vs.* 4CSD	9.07
4CSD *vs.* 6CSD	4.91
2CSA *vs.* 2CSR	4.92
4CSA *vs.* 4CSR	8.26
6CSA *vs.* 6CSR	10.00
2CSA *vs.* 2CSD	11.53
4CSA *vs.* 4CSD	17.32
6CSA *vs.* 6CSD	19.11
2CSR *vs.* 2CSD	6.74
4CSR *vs.* 4CSD	9.21
6CSR *vs.* 6CSD	9.39

CSA, Arabica coffee silverskin; CSR, Robusta coffee silverskin; CSD, decaffeinated coffee silverskin.

Table S3. Average score values obtained for the biscuits through the consumer acceptance test. Results of appearance, odour, taste, flavour, texture and overall liking are reported as score out to 9 (9-point hedonistic scale), whereas those of purchase predisposition as score out to 7 (7-point hedonistic scale).

Attribute	0CS	2CSA	4CSA	6CSA	2CSR	4CSR	6CSR	2CSD	4CSD	6CSD
Appearance	7.50 ± 0.65	7.50 ± 0.51	7.08 ± 0.65	6.50 ± 1.13	6.58 ± 1.51	6.50 ± 1.57	6.17 ± 1.64	7.17 ± 0.91	6.83 ± 1.36	6.67 ± 1.39
Odour	6.67 ± 1.12	6.08 ± 1.33	6.08 ± 0.96	6.00 ± 1.01	5.92 ± 1.33	5.75 ± 1.10	5.33 ± 1.26	6.83 ± 1.15	6.42 ± 0.77	6.08 ± 1.13
Taste	7.42 ± 1.05	6.17 ± 0.91	6.17 ± 1.42	5.83 ± 1.88	6.75 ± 1.18	6.67 ± 1.39	6.08 ± 1.62	7.42 ± 0.96	7.42 ± 1.05	6.83 ± 1.48
Flavour	6.33 ± 1.26	6.08 ± 1.13	5.83 ± 1.53	6.25 ± 1.90	6.17 ± 1.29	6.67 ± 1.51	6.50 ± 1.34	7.25 ± 1.10	7.42 ± 1.20	6.92 ± 1.33
Texture	7.17 ± 1.00	6.50 ± 1.27	6.50 ± 1.13	6.08 ± 1.46	6.67 ± 1.33	6.50 ± 1.52	6.83 ± 1.64	7.50 ± 0.65	7.67 ± 0.86	7.08 ± 1.51
Overall Liking	7.25 ± 0.93	6.25 ± 1.10	6.00 ± 1.30	6.00 ± 1.60	6.50 ± 1.13	6.58 ± 1.27	6.17 ± 1.59	7.33 ± 0.75	7.25 ± 0.93	6.58 ± 1.62
Purchase Interest	5.42 ± 0.50	4.58 ± 0.87	4.42 ± 1.33	4.58 ± 1.33	5.00 ± 1.17	5.08 ± 1.33	4.75 ± 1.31	5.58 ± 0.77	5.75 ± 0.93	4.83 ± 1.48

CSA, Arabica coffee silverskin; CSR, Robusta coffee silverskin; CSD, decaffeinated coffee silverskin.

Table S4. Values (means ± standard deviation) of total phenolic content (TPC) and radical scavenging activity (RSA) of the CS-added biscuits after gastrointestinal digestion. Results of analysis of variance (ANOVA) with Duncan's post hoc test are reported both between different percentages of integration of silverskin (column) and between the different types of silverskin (row).

	% CS	CSA	CSR	CSD	*Significance*
TPC (mg GAE/g)	0	1.49 ± 0.08 [a]	1.49 ± 0.08 [a]	1.49 ± 0.08 [a]	
	2	1.60 ± 0.07 [abA]	1.60 ± 0.02 [bA]	2.04 ± 0.01 [bB]	***
	4	1.70 ± 0.06 [bA]	1.73 ± 0.02 [cA]	2.50 ± 0.01 [cB]	***
	6	1.81 ± 0.01 [cA]	1.88 ± 0.01 [dA]	3.19 ± 0.23 [dB]	***
	Significance	***	***	***	
RSA (μmol TE/g)	0	2.98 ± 0.12 [a]	2.98 ± 0.12 [a]	2.98 ± 0.12 [a]	
	2	3.43 ± 0.00 [bA]	3.29 ± 0.06 [bA]	7.03 ± 0.19 [bB]	***
	4	3.64 ± 0.10 [cA]	3.88 ± 0.05 [cA]	11.12 ± 0.68 [cB]	***
	6	3.80 ± 0.02 [cA]	4.49 ± 0.05 [dB]	13.39 ± 0.36 [dC]	***
	Significance	***	***	***	

CSA, Arabica coffee silverskin; CSR, Robusta coffee silverskin; CSD, decaffeinated coffee silverskin; GAE, gallic acid equivalents; TE, Trolox equivalents. Means followed by the same lower-case (columns) and upper-case (rows) letters are not significant different at $p < 0.05$. Significance: *** = $p < 0.001$.

Table S5. (Pre) and after (Post) in vitro gastrointestinal digestion (GID) of the CS-added biscuits, and bioaccessibility (%), and results of analysis of variance (ANOVA) are reported.

	Sample	Pre-GID *mg GAE/g*	Post-GID *mg TE/g*	*Significance*	Bioaccessibility %
**TPC**	0CS	0.41 ± 0.02	1.49 ± 0.08	***	365.20 ± 31.98
	2CSA	0.42 ± 0.01	1.60 ± 0.07	***	378.41 ± 11.91
	4CSA	0.47 ± 0.01	1.70 ± 0.06	***	361.47 ± 16.92
	6CSA	0.49 ± 0.01	1.81 ± 0.01	***	370.84 ± 3.50
	2CSR	0.41 ± 0.00	1.60 ± 0.02	***	391.92 ± 3.29
	4CSR	0.44 ± 0.01	1.73 ± 0.02	***	392.90 ± 9.60
	6CSR	0.57 ± 0.01	1.88 ± 0.01	***	328.78 ± 4.28
	2CSD	0.72 ± 0.00	2.04 ± 0.01	***	282.32 ± 1.38
	4CSD	1.03 ± 0.03	2.50 ± 0.01	***	244.16 ± 6.87
	6CSD	1.36 ± 0.01	3.19 ± 0.23	***	234.57 ± 16.12
**RSA**	0CS	0.43 ± 0.11	2.98 ± 0.12	***	744.63 ± 255.91
	2CSA	0.93 ± 0.06	3.43 ± 0.00	***	367.97 ± 13.71
	4CSA	1.42 ± 0.05	3.64 ± 0.10	***	256.32 ± 10.56
	6CSA	1.82 ± 0.06	3.80 ± 0.02	***	208.42 ± 7.02
	2CSR	0.21 ± 0.01	2.98 ± 0.12	***	1580.53 ± 68.66
	4CSR	0.34 ± 0.02	3.29 ± 0.06	***	1134.58 ± 80.60
	6CSR	0.71 ± 0.04	3.88 ± 0.05	***	635.00 ± 35.47
	2CSD	1.13 ± 0.01	7.03 ± 0.19	***	624.83 ± 21.68
	4CSD	2.60 ± 0.01	11.12 ± 0.68	***	428.13 ± 27.67
	6CSD	3.40 ± 0.03	13.39 ± 0.36	***	394.23 ± 10.54

CSA, Arabica coffee silverskin; CSR, Robusta coffee silverskin; CSD, decaffeinated coffee silverskin; GAE, gallic acid equivalents; TE, Trolox equivalents. Significance (reported for each biscuit between Pre- and Post- GID): *** = $p < 0.001$.
